# Why not Y naught

**DOI:** 10.1038/s41437-022-00543-z

**Published:** 2022-05-17

**Authors:** Michelle M. Jonika, James M. Alfieri, Terrence Sylvester, Andrew Riley Buhrow, Heath Blackmon

**Affiliations:** 1 Department of Biology, Texas A&M University, College Station, TX USA; 2 Interdisciplinary Program in Genetics and Genomics, Texas A&M University, College Station, TX USA; 3 Interdisciplinary Program in Ecology and Evolutionary Biology, Texas A&M University, College Station, TX USA

**Keywords:** Evolutionary genetics, Evolutionary theory, Population genetics

## Introduction

**Fig. 1:**
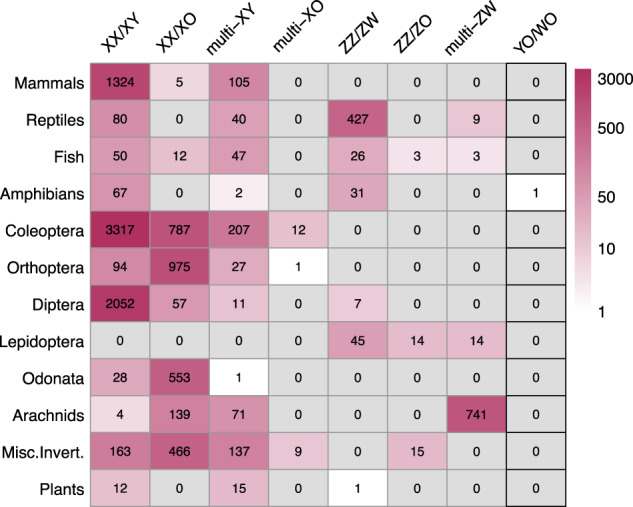
Sex chromosome system distribution among plants and animals. Each cell shows the number of species with a given sex chromosome system. The hue of each cell is based on a log scaling of the count number. Data obtained for this figure were downloaded through open access databases and data included in the following manuscripts: mammals, reptiles, fish, and plants (Tree of Sex Consortium 2014), amphibians (Perkins et al. 2019), Orthoptera (Sylvester et al. 2020), Diptera, Lepidoptera, Odonata, and Coleoptera (Blackmon et al. 2017), arachnids (Araujo et al. 2021, Schneider et al. 2021, Tsurusaki et al. 2021), and misc. invert. (Blackmon et al. 2017).

Genetic sex determination systems have evolved and continue to evolve in a wide diversity of eukaryotes. These are often called sex chromosome systems, even when these chromosomes are homomorphic. In diploid species with male heterogamety, females have two X chromosomes, and males have one X and one Y chromosome (termed XX/XY), but a Y chromosome may be lacking (termed XX/XO systems) (Tree of Sex Consortium 2014). Female heterogametic systems may similarly be ZZ/ZW or ZZ/ZO (Tree of Sex Consortium 2014). Despite the commonness of these sex chromosome systems, incredible variation is present. Synthesizing data from a series of recent papers, we find sex chromosome systems documented in 12,207 plants and animals (Tree of Sex Consortium 2014; Blackmon and Demuth 2015; Blackmon et al. 2017; Perkins et al. 2019; Sylvester et al. 2020; Araujo et al. 2021; Schneider et al. 2021; Tsurusaki et al. 2021; Morelli et al. 2022). Excluding 1453 species with multiple sex chromosomes likely due to sex chromosome-autosome fusions, 7191 (67%) of the remaining 10,754 species exhibit XX/XY systems, and 2994 have XX/XO systems (Fig. 1). In addition, 569 have female heterogametic systems, which is undoubtedly an under-estimate because preparing meiotic spreads for karyotype analysis to identify sex chromosomes is difficult in females, whose ovaries contain fewer cells undergoing meiosis than testes of males.

Among all the species with information in the papers above, only a single species—the New Zealand frog, *Leiopelma hochstetteri*—appears to have a univalent sex-specific chromosome acting as a dominant sex-determining chromosome. We refer to systems like this as YO or WO for male and female heterogamety, respectively. Here, we ask why YO and WO systems are so uncommon. We first evaluate evidence for the existence of YO and WO systems and their potential to arise by reviewing the literature. We then discuss challenges YO and WO systems may face over evolutionary time and the impact of sexually antagonistic (SA) variation on their fates. We conclude that YO and WO systems are unlikely to remain stable, and their transitory nature can explain why they are rare.

## The existence of YO and WO systems

In a YO or WO system, all individuals of the heterogametic sex carry one sex chromosome, which segregates randomly to the gametes, producing a 1:1 sex ratio, with one sex carrying the chromosome as a univalent while the other does not. The only known case is the New Zealand frog, *L. hochstetteri* (Green 1988). In this species, all studied populations have 11 bivalent chromosome pairs and varying numbers of B chromosomes. In the Great Barrier Island population, one of these bivalent pairs has been identified as a largely homomorphic ZW pair, and no B chromosomes have been observed (Green et al. 1993). However, the remaining 11 populations examined all possess a single W chromosome in addition to the 11 bivalents and 0–15 B chromosomes. In these 11 populations, male and female karyotypes consistently differ only by the presence or absence of the univalent W. Various origins for this WO system are possible. Green initially hypothesized a loss of the Z chromosome from a ZW system (Green 1988). However, it seems unlikely that a Z chromosome could be lost, as this requires that it contains no essential genes. It is, therefore, more likely that the ancestral Z had become fixed as a bivalent autosome, similar to the change in Drosophila when the sex-determining role of the dot chromosome was replaced in a turnover event; the turnover involved a former autosome taking control of sex determination, and the heterochromatic dot becoming an autosome (Vicoso and Bachtrog 2013). Furthermore, this hypothesis (Z to autosome transition) is consistent with karyotypic comparisons between the Great Barrier Island and other *L. hochstetteri* populations (Green et al. 1993). One plausible pathway for this transition is the translocation of a dominant female determining allele onto a B chromosome, allowing for the fixation of the Z as an autosome.

At least three fish species may be in intermediate stages of transition to or from a WO or YO system. Two of these are in the genus *Astyanax*. In the Pachón population of the cavefish *Astyanax mexicanus*, a small segregating B chromosome acts as a dominant male determining univalent Y (Imarazene et al. 2021). However, *A. mexicanus* exhibits leaky sex determination. Individuals with a B (possible Y) chromosome still rarely develop as females, and *A. mexicanus* males often have many copies of the same B chromosome. Similarly, in *A. scabripinnis*, a segregating B chromosome (possible W) is found in approximately 30% of the 44 females examined but not in the 20 males studied. However, unlike *A. mexicanus*, the B chromosome in *A. scabripinnis* is a macrochromosome and the karyotype’s second-largest chromosome (Mizoguchi and Martins-Santos 2004). *A. scabripinis’* B macrochromosome may have originated from nondisjunction followed by heterochromatinization, and evidence from other Astyanax species suggests this B chromosome may have evolved in an ancestral lineage (Salvador and Moreira-Filho 1992; Vicente 1994). In the cichlid *Lithochromis rubripinnis*, a B chromosome influencing sex determination occurs more frequently in females than males. Females carrying one copy of this chromosome produce clutches with at least 70% females, and females with two copies produce 100% female clutches (Yoshida et al. 2011).

## The opportunity for YO and WO sex chromosome systems to evolve

One potential impediment to the evolution of YO and WO sex chromosome systems might be difficulty segregating univalent chromosomes. However, as noted above, XO males and ZO females represent 27% of species surveyed, including the large clades Odonata and Orthoptera that are ancestrally XX/XO, suggesting that reliable segregation of univalent chromosomes is possible (Tree of Sex Consortium 2014; Blackmon and Demuth 2015; Blackmon et al. 2017; Perkins et al. 2019; Sylvester et al. 2020). Organisms generally accomplish univalent segregation through one of two broad cell division mechanisms. The first mechanism involves “amphitelic” attachments of spindle fibers to sister kinetochores, connecting them to microtubules from opposite poles, resulting in the segregation of sister chromatids of the univalent sex chromosome during meiosis I (Guerrero et al. 2010). Alternatively, “syntelic” attachments form with sister kinetochores connected to microtubules from a single spindle pole, resulting in a mono-oriented chromosome, with sister chromatids of the univalent sex chromosome segregating during meiosis II (Guerrero et al. 2010). Organisms vary in the cell phase during which attachments occur and which organelles are associated (Fabig et al. 2016).

Furthermore, the general lability of sex chromosome systems illustrates the frequent incorporation of new genome regions into the sex chromosomes and the loss of existing sex chromosomes. The loss of Y(W) chromosomes is well-documented in Lepidoptera, Nematoda, Orthoptera, and Odonata, with some groups such as Coleoptera having many independent Y chromosome losses (Kiauta 1969; Bull 1983; Traut et al. 2007; Blackmon and Demuth 2014), including several cases in mammals (in mole voles and spiny rats, see Just et al. 1995, 2007; Arakawa et al. 2002). Some clades have both XO and XY systems. For example, in Polyneoptera (an insect clade including the orders Blattodea, Dermaptera, Embiidina, Mantodea, Notoptera, Orthoptera, Phasmatodea, and Plecoptera), 17 genera have both XO and XY systems, and 6 have both XO and Multi-XY systems. The ancestral system of Polyneoptera is likely XO, and the 23 transitions from XO to XY and from XO to multi-XY sex chromosome systems are certainly underestimated (Sylvester et al. 2020). There are examples of other sex chromosome changes in insects. For instance, the sex chromosomes of *Drosophila melanogaster* likely represent a transition from an ancestral system where the dot chromosome (now an autosome) functioned as the X, and the current sex chromosomes were autosomes (Vicoso and Bachtrog 2013). Other non-canonical sex chromosome origins are suggested in Lepidoptera and Muscid flies (Fraïsse et al. 2017; Meisel et al. 2020). These examples of sex chromosome system lability indicate the possibility for YO or WO systems to arise by incorporating new genomic regions and loss of existing sex chromosomes.

Another possibility for the non-canonical origins of sex chromosomes involves B chromosomes. B and Y/W chromosomes are often highly repetitive and gene-poor. There are several potential ways a B chromosome could gain a sex-determining factor. The first possibility is the transposition of the ancestral male-determiner to a B chromosome from the Y chromosome. Similar events have been documented with transposition onto autosomes (Vicoso and Bachtrog 2013; Meisel et al. 2020; Pan et al. 2021). The second possibility is for a new male-determiner to arise on a B chromosome. One example of the recruitment of B chromosomes for sex determination is observed in three Lepidopterans. The W chromosomes in these insects correspond to a B chromosome gaining a femaleness factor (Fraïsse et al. 2017). In addition, in the Lepidoptera species *Dryas iulia*, a W chromosome has been suggested to be a captured B chromosome (Lewis et al. 2021). The recruitment of B chromosomes in these examples is supported rather than canonical models of Z-autosome fusion or sex chromosome turnover or other non-canonical models such as the B chromosome fusion hypothesis that led to a giant sex chromosome in Cichlid fish (Fraïsse et al. 2017; Lewis et al. 2021; Conte et al., 2021). B chromosomes are common across all well-studied major eukaryote taxa, including 2087 plant species, 736 animal species, and 14 fungi species (D’Ambrosio et al. 2017), suggesting that opportunities for such origins of YO or WO systems could exist.

## The challenges to YO or WO sex chromosome systems maintenance

SA genes are polymorphic for alleles that benefit one sex at the expense of the other (Fisher 1931; Charlesworth and Charlesworth 1980) and may be involved in sex chromosome origin and evolution (Charlesworth 1991; Otto et al. 2011). Various empirical and theoretical studies support the view that SA mutations frequently occur (Rice 1987; Innocenti and Morrow 2010; Ironside 2010; Connallon and Clark 2014; Anderson et al. 2020). SA polymorphisms may limit the stability of YO or WO systems. If SA loci are present on autosomes, fusions with a univalent Y or W chromosome would be favored if they create a linkage between the SA locus and the sex-determining locus (Charlesworth and Charlesworth 1980; van Doorn and Kirkpatrick 2007). The Japan Sea stickleback is one well-investigated example where chromosome 9 is fused to the Y chromosome (Kitano et al. 2009). Empirical evidence suggests that the frequent transitions from XX/XO to XX/XY in Polyneoptera involve X-autosome fusion (Sylvester et al. 2020). If similar fusions occur in YO or WO systems, they will lead to transitions into XY or ZW systems, respectively. This process may be one reason for the rarity of YO and WO systems.

A second challenge to maintaining a univalent sex chromosome is a lack of recombination. Lack of recombination in a genome region is associated with loss of sequence integrity. Transposable elements will rapidly expand within non-recombining regions (Charlesworth et al. 1994; Bachtrog 2005; Nozawa et al. 2021). Reduced effective population size allows several processes leading to the loss of gene function, slower adaptation, and eventually gene loss (reviewed by Charlesworth and Charlesworth 2000; Steinemann and Steinemann 2005; Bachtrog 2008). This process often leads to a reduction in the size of the Y chromosome and potentially to eventual loss of all functional content on a sex chromosome and Y or W chromosome loss (Hjelmen and Johnston 2017). Nevertheless, strong purifying selection can maintain essential genes in non-recombining Y-linked regions. For instance, the mammalian SRY gene has survived in the vast majority of mammals for the last 150 million years, despite not recombining with its X homolog (Veyrunes et al. 2008).

The decay of functional genic content in non-recombining genome regions may also favor a role for B chromosomes in the origins of YO/WO systems due to their small size. Briefly, a new univalent sex chromosome must increase in frequency and fix in the heterogametic sex. However, degeneration due to lack of recombination will decrease the fitness contribution of the univalent chromosome. The rate at which the fitness contribution decreases will scale with the number of sites under selection. Successful univalent sex chromosomes would thus be predicted to be small. This relationship between size and fixation probability for non-recombining sex chromosomes has been suggested to explain the paucity of sex chromosome-autosome fusions observed in the genus Drosophila (Anderson et al. 2020).

## Conclusion

Synthesizing the above observations, we propose that YO and WO sexual systems may frequently arise across the tree of life, but both sexual antagonism and mutational decay may lead them to be inherently unstable, transitory, and unlikely to fix in populations. *Astyanax mexicanus* and closely related species may help us understand how univalent sex chromosomes evolve over short time periods and how their gene content evolves, including the possible role of B chromosomes in sex chromosome evolution.
